# Up-Regulation of Cyclooxygenase-2 (COX-2) Expression by Temozolomide (TMZ) in Human Glioblastoma (GBM) Cell Lines

**DOI:** 10.3390/ijms23031545

**Published:** 2022-01-28

**Authors:** Francesca Lombardi, Francesca Rosaria Augello, Serena Artone, Mitilda Karoli Gugu, Maria Grazia Cifone, Benedetta Cinque, Paola Palumbo

**Affiliations:** Department of Life, Health & Environmental Sciences, University of L’Aquila, Building Delta 6, Coppito, 67100 L’Aquila, Italy; francesca.lombardi@univaq.it (F.L.); francescarosaria.augello@graduate.univaq.it (F.R.A.); serena.artone@student.univaq.it (S.A.); mitilda.gugu@graduate.univaq.it (M.K.G.); mariagrazia.cifone@univaq.it (M.G.C.)

**Keywords:** glioblastoma, COX-2, temozolomide, COX-2 inhibitor, NS398, T98G, U251MG, β-catenin, MGMT, SOX-2

## Abstract

TMZ-resistance remains a main limitation in glioblastoma (GBM) treatment. TMZ is an alkylating agent whose cytotoxicity is modulated by O6-methylguanine-DNA methyltransferase (MGMT), whose expression is determined by MGMT gene promoter methylation status. The inflammatory marker COX-2 has been implicated in GBM tumorigenesis, progression, and stemness. COX-2 inhibitors are considered a GBM add-on treatment due to their ability to increase TMZ-sensitivity. We investigated the effect of TMZ on COX-2 expression in GBM cell lines showing different COX-2 levels and TMZ sensitivity (T98G and U251MG). β-catenin, MGMT, and SOX-2 expression was analyzed. The effects of NS398, COX-2 inhibitor, alone or TMZ-combined, were studied evaluating cell proliferation by the IncuCyte^®^ system, cell cycle/apoptosis, and clonogenic potential. COX-2, β-catenin, MGMT, and SOX-2 expression was evaluated by RT-PCR, Western blotting, and immunofluorescence and PGE2 by ELISA. Our findings, sustaining the role of COX-2/PGE2 system in TMZ-resistance of GBM, show, for the first time, a relevant, dose-dependent up-regulation of COX-2 expression and activity in TMZ-treated T98G that, in turn, correlated with chemoresistance. Similarly, all the COX-2-dependent signaling pathways involved in TMZ-resistance also resulted in being up-modulated after treatment with TMZ. NS398+TMZ was able to reduce cell proliferation and induce cell cycle arrest and apoptosis. Moreover, NS398+TMZ counteracted the resistance in T98G preventing the TMZ-induced COX-2, β-catenin, MGMT, and SOX-2 up-regulation.

## 1. Introduction

Glioblastoma (GBM) represents the most frequent and aggressive primary malignancy of the central nervous system in adults. Despite advances in therapy, the patient outcome remains poor mainly due to the development of resistance to conventional therapies [1,2], such as the chemotherapy approach with the oral alkylating agent temozolomide (TMZ) [3,4]. TMZ exerts its cytotoxic effect by alkylating the genomic DNA at the N7 and O6 positions of guanine, resulting in mutations or DNA cross-linking and cell cycle arrest [5]. O6-methylguanine-DNA methyltransferase (MGMT) is a key enzyme able to directly remove alkyl groups from the O6 position of guanine, whose expression is determined by the methylation status of the MGMT gene promoter and is considered a significant predictor in TMZ response [6]. High levels of MGMT or lack of MGMT promoter methylation are associated with TMZ-resistance and poorer prognosis in GBM patients [7,8].

Cyclooxygenase-2 (COX-2), the inducible isoform of prostaglandin H synthase, a pro-inflammatory enzyme, is often up-regulated in GBM cells and tissues, and higher COX-2 expression is associated with most malignant histological grade [9,10]. Additionally, COX-2 protein is constitutively expressed in human adherent GBM cell lines [9,11,12], as well as in derived tumorspheres [13]. The glioma-promoting effects of COX-2 induction are mainly associated with its product, prostaglandin E2 (PGE2) [14], which, in turn, at high levels, favors tumor initiation and progression and is also implicated in the development of therapy resistance [15,16,17]. In GBM cell lines and mouse models, COX-2-derived PGE2 promotes glioma stem cells (GSC) self-renewal and therapy resistance through the EP4 receptor that activates the MAPK signaling cascade, leading to the up-regulation of the inhibitor of differentiation 1 (Id1), a main mediator that abrogates differentiation signals in GSC and contributes to chemoresistance in GBM. Moreover, Id1 could also regulate stemness through Wnt signaling, and when Id1 was genetically or pharmacologically inhibited, the cytotoxic effect of TMZ was enhanced in vitro, and the survival in a GBM xenograft model was extended [18,19].

Several in vitro studies have described the ability of the selective COX-2 inhibitors to improve GBM sensitiveness to traditional chemo- and radiotherapy, increasing cell death and apoptosis and reducing tumor migration and stemness potential [20,21,22]. The efficacy of this combination was also highlighted in studies on animal models. The COX-2 inhibition, combined with TMZ, significantly enhanced the cytotoxic drug effect in a mouse GBM orthotopic xenograft, improving the mean survival rate [23,24].

A retrospective clinical study in recurrent GBM showed six months progression-free survival of 43% of patients treated with TMZ at low-dose plus celecoxib, as opposed to the 21% treated with standard TMZ maintenance therapy [25]. The COX-2 inhibitor celecoxib was also included as part of the “CUSP9” treatment protocol as one of nine drugs inhibiting growth-enhancing pathways of GBM [26]. Evidence shows that COXIBs, through COX-2 activity reduction, lead to the Wnt/β-catenin signaling inhibition, thus preventing chemoresistance on GBM cell lines [27]. Wnt/β-catenin signaling, whose activation has been shown to be strongly involved in the development of glioma, in the maintenance of stem potential, invasiveness, angiogenesis, radio, and chemoresistance, is considered one of the best-known pathways mainly targeted by COX-2 inhibition in GBM [24,28]. NSAIDs, including sulindac, exisulind, and celecoxib, are known to inhibit Wnt signaling [29], decrease β-catenin levels, and then inhibit the transcriptional activity of the β-catenin/T-cell factor (TCF)/lymphoid enhancer factor (LEF) complex [30]. PGE2 promotes the β-catenin signaling through mechanisms that require the Gαs binding to axin and the phosphorylation of GSK-3β via AKT [17,31].

Wickstrom et al. reported that Wnt/β-catenin pathway activation was significantly associated with drug resistance due to the induction of MGMT expression. The Authors show evidence that the genetic or COXIB-induced pharmacological inhibition of Wnt/β-catenin signaling restored chemosensitivity in GBM by down-regulating MGMT [27].

Of interest, TMZ has been shown to influence many cellular signaling pathways linked in the GBM-chemoresistance, including Wnt/β-catenin, MGMT, and stemness properties [32,33,34,35].

The sulphonamide derivative NS398 is a highly selective COX-2 inhibitor, and even if not yet approved by the Food and Drug Administration (FDA) for clinical use, several studies have reported effective activity on glioma cell lines [20,21]. NS398 has been reported to significantly reduce the proliferation and migration rate and alter the cell cycle phase distribution of GBM cell lines [20,36].

Our group recently reported evidence that NS398 strongly influenced the cell proliferation and migration rate of adherent U87MG and T98G cell lines and cell growth and morphologic features in GSC derived from adherent GBM cells. The inhibitor induced the autophagy pathway both in GSC and adherent U87MG and T98G cell lines [13]. In addition, the COX-2 inhibitor led to a functional modification of extracellular vesicles (EVs) released by the relative derived GSC. Indeed, EVs secreted by NS398-treated GSC, obtained from either U87MG or T98G cells, significantly inhibited cell migration and induced autophagy in recipient adherent U87MG and T98G cells, thus leading to effects quite comparable with those directly produced by the inhibitor in the same cells. These findings showed that NS398 influenced both GBM cell lines likewise, despite their different COX-2 expression levels, as well as the intrinsic genetic diversity, including TP53 gene status, MGMT activity, base excision repair (BER), or BRCA1 pathways, associated with their different TMZ-sensitivity/resistance [37,38,39]. Some groups stated that the effects of COXIBs on GBM cells or GSC isolated from primary GBM cultures, including counteracting TMZ-resistance, could be independent of COX-2 expression and activity and ascribed to the interaction with other targets than COX-2, including the Wnt/β-catenin pathway [12,40].

Moved by these findings and suggestive hypotheses, in the present study, we wanted to evaluate the ability of TMZ to influence COX-2 expression and activity in two GBM cell lines, i.e., T98G and U251MG, showing different sensitivity to TMZ. Moreover, given the involvement of the Wnt/β-catenin pathway in GBM chemoresistance and stemness potential, we evaluated the β-catenin, MGMT, and SOX-2 expression levels. Afterward, the ability of NS398 to overcome TMZ-resistance was studied on T98G as a single agent or in combination with TMZ. In addition, we evaluated the behavior of the COX-2 negative U251MG cell line under the same experimental conditions to check whether the mechanism behind the NS398 effects could be COX-2-independent. Finally, in the attempt to delineate the potential mechanisms involved in the intricate framework of the assumed COX-2-dependent TMZ-resistance of GBM cells, we investigated the relationships between COX-2/PGE2 system, β-catenin, MGMT, and stemness potential.

## 2. Results

### 2.1. COX-2 Expression and TMZ Sensitivity of GBM Cell Lines

**Figure 1:**
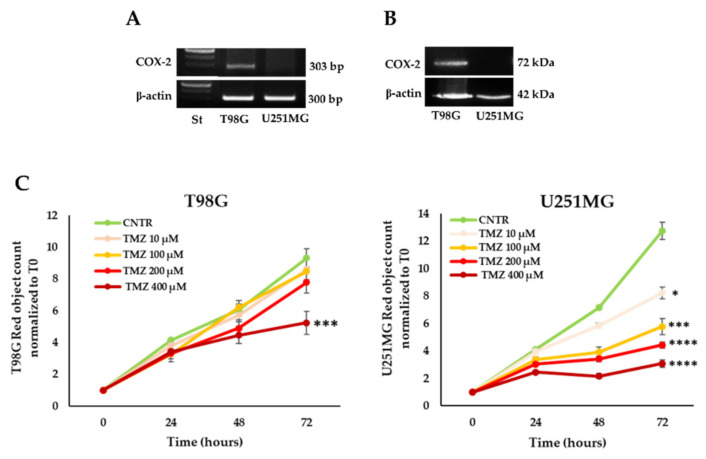
COX-2 expression and TMZ sensitivity of GBM cell lines. COX-2 expression was analyzed in T98G and U251MG cells by (**A**) reverse transcriptase PCR and (**B**) WB. Images from one representative out of three independent experiments are shown. “St” is a DNA ladder (100 bp); β-actin was used as the internal control. (**C**) Growth curves of T98G and U251MG cells were obtained through IncuCyte^®^ Live Cell Imager. Cell cultures were treated until 3 days with TMZ (10–400 μM) and stained using the NucLight Rapid Red reagent. The results, relative to one representative out of three experiments performed in triplicate, are expressed as mean ± SD. For comparative analysis of groups of data, two-way repeated-measures ANOVA followed by Dunnett’s post hoc test was used (* *p* < 0.05, *** *p* < 0.001, **** *p* < 0.0001 vs. CNTR).

First, we wanted to verify the expression status of COX-2 in two GBM cell lines: T98G and U251MG. Either reverse transcriptase PCR (Figure 1A) or Western blot (WB) assay (Figure 1B) showed the COX-2 constitutive expression in T98G and the lack of COX-2 in U251MG, thus confirming previous findings [11,12,41]. Afterward, the effect of TMZ on cell proliferation was evaluated on the two GBM cell lines, exposed to increasing concentrations of drug (10–400 µM) or drug vehicle DMSO (CNTR) by IncuCyte^®^ Live Cell Imager instrument every 4 h for a total timeline of 3 days (72 h). The T98G cell proliferation, as expected, was significantly affected only at the highest concentration of TMZ (400 µM), thus confirming its elevated chemoresistance (Figure 1C). The U251MG cells showed a significant reduction in proliferation compared with CNTR cells at all concentrations of TMZ (Figure 1C).

### 2.2. Effect of TMZ on COX-2 Expression in GBM Cell Lines

With the aim of analyzing whether TMZ was able to influence the COX-2 expression in GBM cells, the T98G and U251MG cells were treated with the different concentrations of TMZ for 3 days, and the COX-2 levels were analyzed.

**Figure 2:**
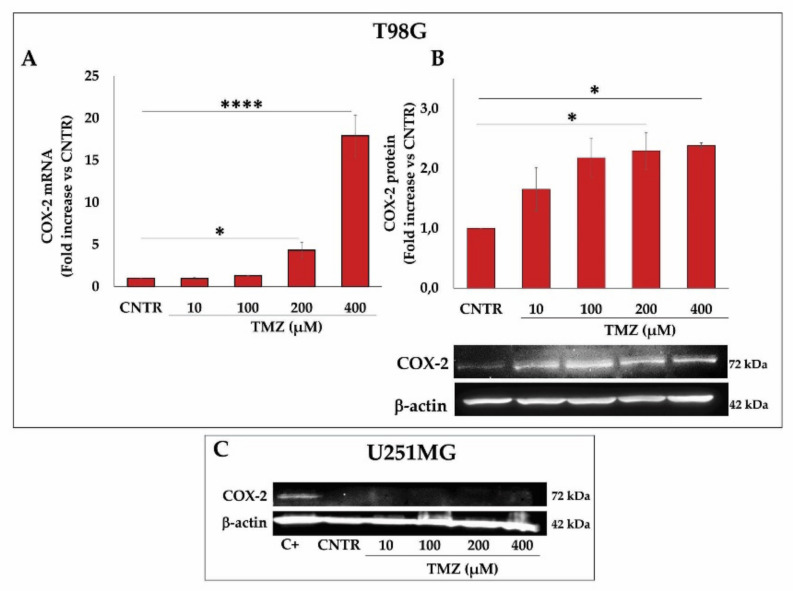
Effect of TMZ on COX-2 expression in GBM cell lines. T98G and U251MG were exposed to increasing concentrations of TMZ (10–400 µM) for 3 days, and COX-2 levels were assayed. (**A**) Real-time PCR results are expressed as mean ± SEM of two independent experiments performed in triplicate and are shown as fold vs. CNTR. (**B**,**C**) Immunoblotting assay for COX-2 was performed on T98G and U251MG in the presence of vehicle (CNTR) or TMZ at indicated concentrations. Densitometric analysis is presented normalizing vs. β-actin. Data from three independent experiments are expressed as mean ± SEM. Representative images of three independent WBs are presented. C+ = positive control (not treated T98G cells). For comparative analysis of groups of data, one-way ANOVA followed by Dunnett’s post hoc test was used (* *p* < 0.05, **** *p* < 0.0001 vs. CNTR).

In T98G cells, the COX-2 mRNA levels were significantly increased after exposure with TMZ at 200 µM and enhanced dramatically at 400 µM when compared with CNTR cells (Figure 2A). In addition, WB assay revealed that the COX-2 protein levels were significantly enhanced at increasing TMZ concentrations, resulting in being significant at concentrations of TMZ ≥ 200 µM with respect to CNTR (Figure 2B). In the COX-2 negative (COX-2^-^) GBM cell line, U251MG, TMZ failed to induce the COX-2 expression at all tested concentrations (Figure 2C).

### 2.3. Influence of TMZ Treatment on β-Catenin, MGMT, and SOX-2 Levels in GBM Cells

**Figure 3:**
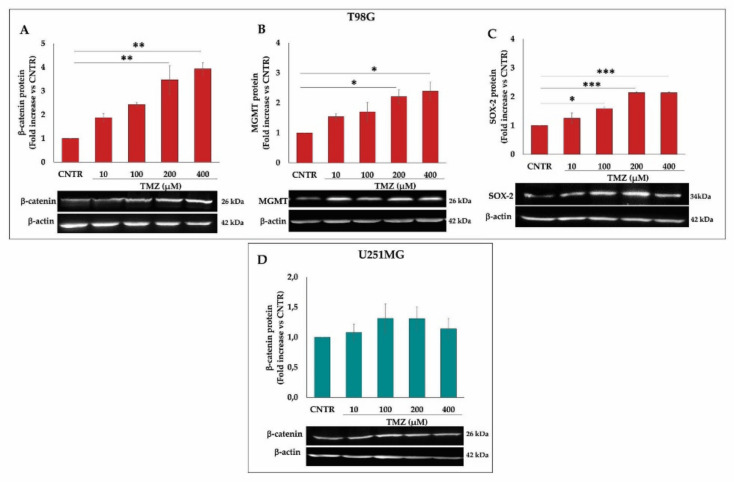
Influence of TMZ treatment on β-catenin, MGMT, and SOX-2 levels in GBM cells. GBM cell lines, T98G and U251MG, were treated with increasing concentrations of TMZ (10–400 µM) for 3 days. (**A**,**D**) Immunoblotting assays for β-catenin are shown. (**B**) MGMT and (**C**) SOX-2 expression levels were detected by immunoblotting assay in T98G cells. Densitometric analysis is presented normalizing vs. β-actin. The data from three independent experiments are shown as the mean ± SEM and expressed as fold increase vs. CNTR. Images from one representative out of three independent experiments are shown. For comparative analysis of groups of data, one-way ANOVA followed by Dunnett’s post hoc test was used (* *p* < 0.05, ** *p* < 0.01, *** *p* < 0.001 vs. CNTR).

Based on the obtained results on TMZ-resistant T98G regarding the up-regulation of COX-2 by TMZ, we wanted to verify whether TMZ was able to modulate the key proteins closely associated with the activity of COX-2 and strongly implicated in the progression and resistance of GBM. The effects of TMZ on the β-catenin, MGMT, and SOX-2 expression were evaluated after exposure to increasing doses of the alkylating agent (10–400 µM) for 3 days. The results of these experiments show an up-modulation of β-catenin proportional to the increasing concentrations of TMZ in T98G, resulting in being significant at TMZ ≥ 200 µM (Figure 3A). Similarly, the MGMT levels were enhanced accordingly to increasing TMZ concentrations in T98G cells, being significant at 200 and 400 µM (Figure 3B). To assess the effects of TMZ on the stemness potential of T98G cells, we also analyzed SOX-2 levels. A significant and dose-dependent increase in the SOX-2 protein expression was detected at 100 µM, 200 µM, and 400 µM with respect to CNTR (Figure 3C). On the other hand, TMZ did not affect the β-catenin expression in the U251MG cells (Figure 3D). In the MGMT negative (MGMT^-^) cell line U251MG, the MGMT, as well as SOX-2 expression, was not influenced by TMZ treatment for 3 days (data not shown).

### 2.4. Effects of NS398 on PGE2 Release and Cell Proliferation Rate in GBM Cell Lines

**Figure 4:**
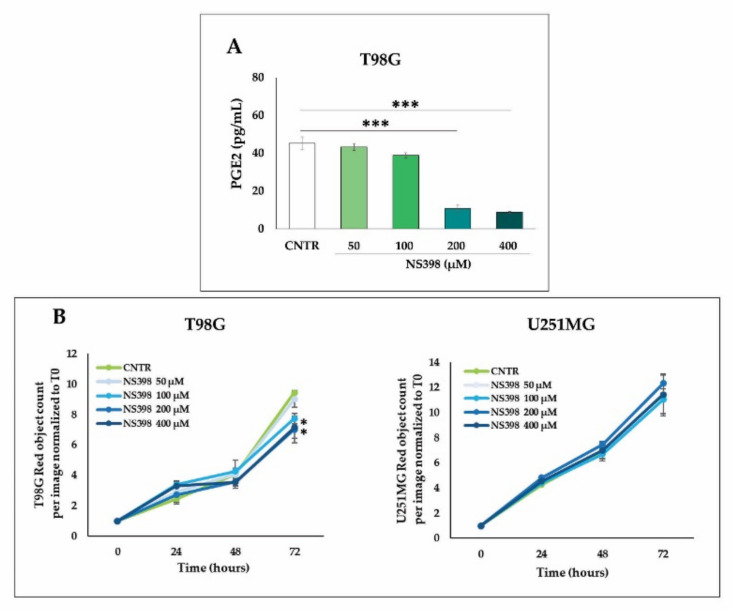
Effects of NS398 on PGE2 release and cell proliferation rate on GBM cell lines. (**A**) PGE2 levels in supernatants of NS398-treated T98G cells were assayed by ELISA. Results are expressed as mean ± SEM of two experiments performed in duplicate. The one-way ANOVA for repeated measures followed by Dunnett’s post hoc test (*** *p* < 0.001 vs. CNTR) was used. (**B**) Effect of NS398 on the cell proliferation rate of T98G and U251MG treated or not (CNTR) for 3 days with NS398 (50–400 μM) was evaluated. Cells were stained with NucLight Rapid Red reagent to detect live and healthy cells, and growth curves of GBM cell lines were analyzed through IncuCyte^®^ Live Cell Imager. The results, relative to one representative out of three experiments performed in triplicate, are expressed as mean ± SD. For comparative analysis of groups of data, two-way repeated-measures ANOVA followed by Dunnett’s post hoc test was used (* *p* < 0.05 vs. CNTR).

The effect of NS398 on the PGE2 levels, related to COX-2 activity, was evaluated in the supernatant of cell cultures using different concentrations (50–400 µM) for 3 days. In T98G cells, the NS398 strongly inhibited the COX-2 activity at higher concentrations (200 and 400 µM) (Figure 4A). As expected, PGE2 levels were undetectable in the culture medium of U251MG cells (data not shown). To identify the most appropriate concentration of COX-2 inhibitor to use in the next experiments, GBM cells were treated with increasing concentrations of NS398 (50–400 µM) to continuously evaluate the cell growth by IncuCyte^®^ Live Cell Imager instrument every 4 h for a total timeline of 3 days (72 h). The NS398 exposure influenced the proliferation rate of T98G COX-2-positive (COX-2^+^) cell line showed significant differences compared with CNTR cells at maximal concentrations, 200 and 400 µM (Figure 4B). Of note, treatment with NS398, even at the highest concentrations, had no effect on cell proliferation of U251MG (Figure 4B). Based on the obtained data, the NS398 concentration of 200 µM, being able to effectively inhibit COX-2 activity as well as proliferation rate of T98G cells, was chosen for the subsequent experiments designed to analyze the effects of combined treatment NS398+TMZ. In addition, the results obtained with U251MG, being COX-2^-^cells, confirmed the specificity of the NS398 inhibitor.

### 2.5. Effects of NS398 Alone or Combined with TMZ on GBM Cell Viability, Cell Cycle Distribution, and Apoptosis

**Figure 5:**
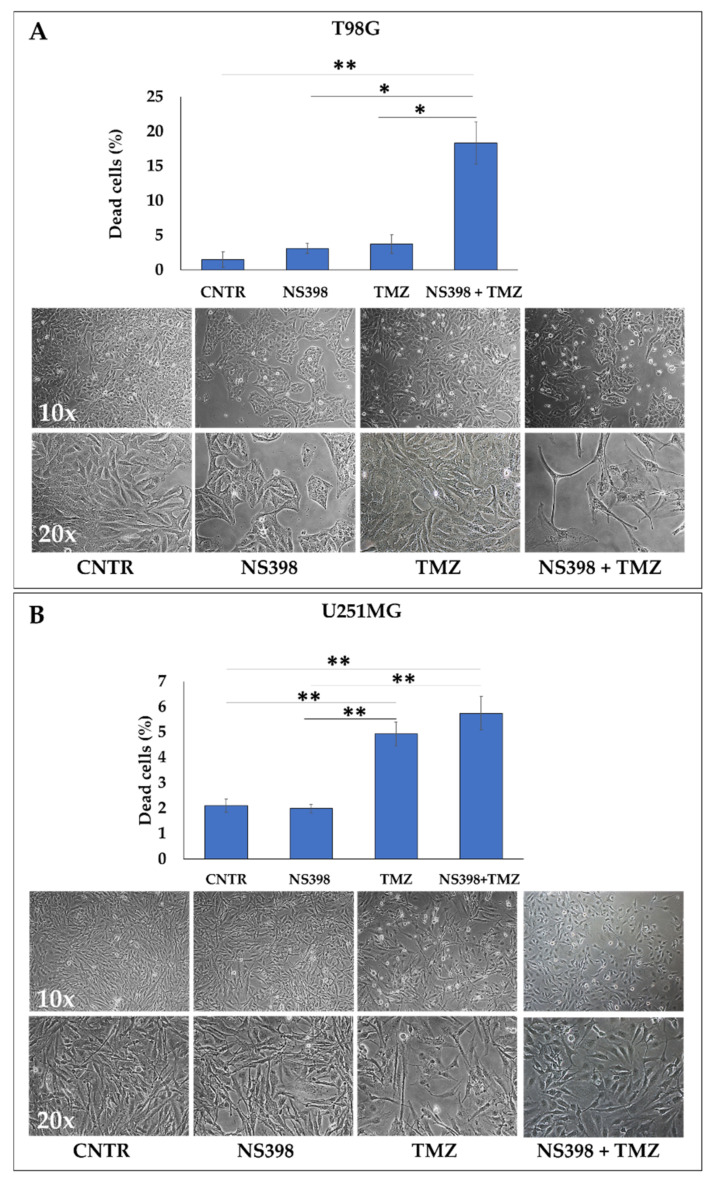
Effects of NS398 alone or combined with TMZ on GBM cell viability. (**A**) T98G and (**B**) U251MG cells were incubated for 3 days in the absence (CNTR) or presence of NS398 (200 µM), TMZ (200 µM for T98G, and 10 µM for U251MG) and their combination. The cell viability was evaluated by Trypan blue staining and data expressed as dead cell percentage values (mean ± SEM). The experiments were repeated at least three times in triplicate. For comparative analysis of groups of data, one-way ANOVA followed by Tukey post hoc test was used (* *p* < 0.05, ** *p* < 0.01). Representative phase-contrast microscopic images from one out of three independent experiments are shown (10× and 20× magnification).

To verify the effect of the COX-2 inhibitor, NS398, on TMZ-resistance, the GBM cell lines were exposed to single agents and their mixture for 3 days. Based on the above results, the chosen TMZ concentrations to keep the proliferation rate above 50% were 200 µM for T98G cells and 10 µM for U251MG cells. The biological parameters such as viability, cell cycle, and apoptosis levels were evaluated. In the T98G cell line, the single agents, NS398 and TMZ, did not induce a significant increase in dead cell percentage when compared with CNTR (Figure 5A). The TMZ exposure significantly enhanced the dead cell percentage only in U251MG, thus confirming the high chemoresistance of T98G. The co-treatment (NS398+TMZ) induced a significant increase in cell mortality in T98G, showing a synergistic effect (Figure 5A); on the contrary, the combination induced an effect similar to TMZ alone in COX-2^-^ U251MG, as reported in Figure 5B. Representative images from microscopic observations of the cell cultures under the different treatment conditions, displayed in the below panels of Figure 5, confirmed the reduction in cell proliferation rate.

**Figure 6:**
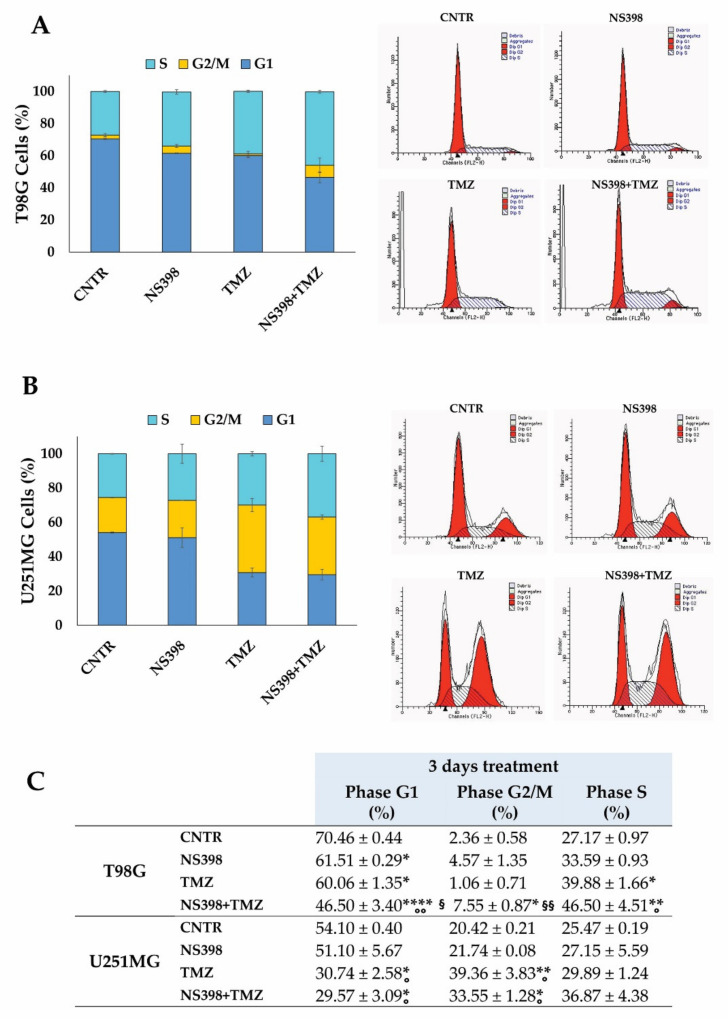
Effects of NS398 alone or combined with TMZ on cell cycle distribution of GBM cell lines. (**A**,**B**) GBM cells were exposed to NS398, TMZ, and NS398+TMZ for 3-day culture, and the analysis of cell cycle phases of T98G and U251MG cells was performed by flow cytometry. (**C**) Values of flow cytometric histograms showing percentage distribution of cycle phases referring to two independent experiments in duplicate are graphed and reported (mean ± SEM). For comparative analysis of groups of data, a one-way analysis of variance (ANOVA) with post hoc Tukey test was used (* *p* < 0.05, ** *p* < 0.01, **** *p* < 0.0001 vs. CNTR; ° *p* < 0.05, °° *p* < 0.01 vs. NS398; § *p* < 0.05, §§ *p* < 0.01 vs. TMZ). The flow cytometric profiles from a representative experiment are also shown.

The effect of TMZ, NS398, and their combination on cell cycle distribution by flow cytometry was analyzed on T98G and U251MG. The cell cycle analysis of T98G cells showed that NS398 treatment significantly reduced the percentage of T98G cells in G1. Meanwhile, the presence of TMZ determined not only an evident decrease in cells in G1 but also a clear increase in the S-phase. Of interest, the NS398+TMZ combination altered the cell cycle phase distribution, inducing a significant reduction in G1-phase with respect to CNTR, NS398, and TMZ, with a significant cell accumulation in G2/M-phase with respect to CNTR and TMZ (Figure 6A,C). In the U251MG cells, the treatment with TMZ caused similar effects as the combination, resulting in a reduction in the cell population in the G1 phase, with a clear increase in the G2/M phase fraction when compared with CNTR and NS398-treated cells at 3 days (Figure 6B,C).

**Figure 7:**
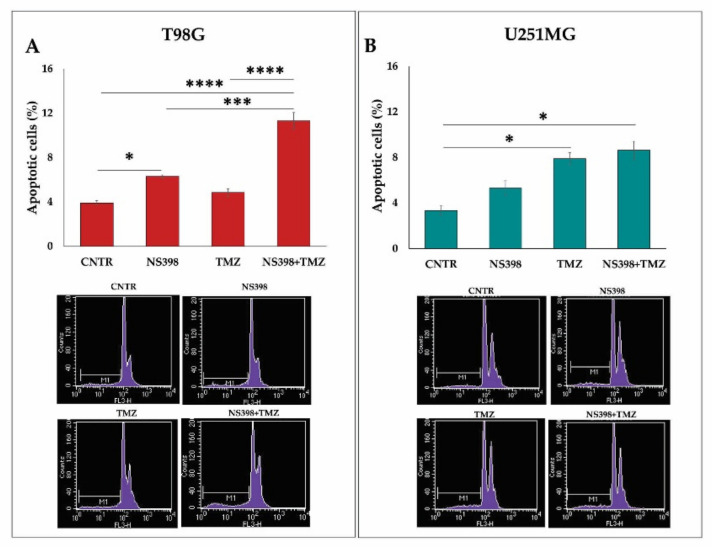
Effect of NS398, alone or combined with TMZ, on GBM cell apoptosis. (**A**,**B**) GBM cell lines were exposed to the NS398 and TMZ, alone or in combination for 3 days, and flow cytometry analysis was performed to detect the apoptotic cells in T98G and U251MG. Histograms show the results from two independent experiments in duplicate expressed as mean ± SEM. The ANOVA one-way test followed by Tukey’s test for multiple comparisons was performed (* *p* < 0.05, *** *p* < 0.001, **** *p* < 0.0001). Flow cytometric profiles from one representative experiment are also shown.

A moderate increase in apoptotic cell percentage, detected by the sub-G1 peak, in T98G treated for 3 days with the inhibitor NS398 alone was registered. On the other hand, the exposure for 3 days to TMZ alone failed to induce significant levels of apoptosis. Of note, the addition of NS398+TMZ led to a much higher and statistically significant level of apoptosis than all other culture conditions (Figure 7A). In U251MG, NS398, which alone did not increase the sub-G1 population, when combined with TMZ, did not significantly influence the apoptotic cell percentage observed after the treatment with TMZ alone (Figure 7B). Overall, in the U251MG cell line, COX-2^-^, the COX-2 inhibition by NS398 did not strengthen the antiproliferative, apoptotic, and cytostatic effect of TMZ. Otherwise, in COX-2^+^, TMZ-resistant T98G cells, the reduction in COX-2 expression and activity led to the appearance of susceptibility to TMZ, resulting in a significant cell proliferation reduction, cell cycle arrest, and increased apoptosis level, suggesting the involvement of COX-2 activity underlying the chemoresistance mechanisms.

### 2.6. Effects of NS398 Alone or Combined with TMZ on Colony Formation Ability of GBM Cell Lines

**Figure 8:**
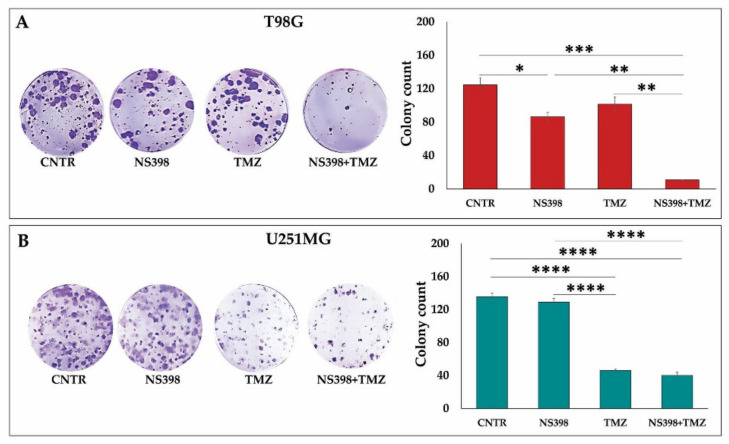
Effects of NS398 alone or combined with TMZ on colony formation ability of GBM cell lines. (**A**) T98G and (**B**) U251MG, untreated (CNTR) and treated for 3 days with NS398 and TMZ, alone or in combination, were cultured until the colony formation (~20 days for T98G and ~18 days for U251MG cells). Representative microscopic images of generated colonies are shown. Colonies formed were counted using the ColonyCountJ program, and quantitative results are expressed as total colony counts from three independent experiments in duplicate (mean values ± SEM). For comparative analysis of groups of data, one-way ANOVA followed by Tukey post hoc test was used (* *p* < 0.05, ** *p* < 0.01, *** *p* < 0.001, **** *p* < 0.0001).

NS398 alone significantly affected the colony generation in the COX-2^+^ cell line, T98G (Figure 8A). As expected, the number of colonies generated by T98G cells was not significantly affected by TMZ standalone (Figure 8A). Notably, the combination NS398+TMZ dramatically inhibited the clonogenic potential in T98G cells. No effect was observed in U251MG cells when exposed to NS398 compared with CNTR, while very few colonies were developed after treatment with TMZ (Figure 8B). Additionally, in the clonogenic ability, no significant difference was detected between NS398+TMZ combination and TMZ alone treatment in U251MG (Figure 8B).

### 2.7. NS398 Counteracted TMZ-Induced COX-2 Overexpression in T98G Cells

**Figure 9:**
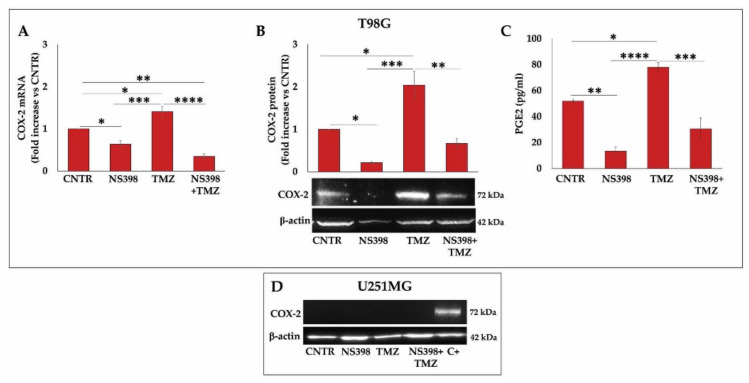
NS398 counteracted TMZ-induced COX-2 overexpression in T98G cells. (**A**) The SYBR-Green Real-Time PCR analysis of the COX-2 gene was performed in T98G cells incubated for 3 days with NS398 and TMZ, alone or in combination. mRNA levels were relative to the amount of GAPDH mRNA. Data from three experiments in duplicate (mean ± SEM) are shown as fold increase vs. untreated cells (CNTR). (**B**,**D**) Immunoblotting assay for COX-2 was performed on T98G and U251MG. C+ = positive control (not treated T98G). Densitometric analysis is presented normalizing vs. β-actin. The data from three independent experiments are shown as the mean ± SEM and expressed as fold increase vs. CNTR. Images from one representative out of three independent experiments are shown. (**C**) PGE2 levels were assayed in cell supernatants of T98G by ELISA. Results are expressed as mean ± SEM and are relative to two experiments performed in duplicate. For comparative analysis of data, the one-way ANOVA followed by Tukey post hoc test was performed (* *p* < 0.05; ** *p* < 0.01; *** *p* < 0.001; **** *p* < 0.0001 vs. CNTR).

The COX-2 gene expression was assessed through real-time PCR in GBM cells after a 3-day treatment with single agents or their combination, and results are presented in Figure 9A. As reported above, the TMZ exposure induced a relevant and significant COX-2 gene expression in the T98G cell line. Of note, NS398, which alone led to a downmodulation of the basal COX-2 levels, when added together with TMZ, was able to counteract the TMZ-induced COX-2 overexpression in this cell line, revealed by COX-2 mRNA and protein level assay (Figure 9A,B). PGE2 levels’ assay in T98G cell supernatants also confirmed these results, being the PGE2 production affected by treatment in an overlapping way (Figure 9C). On the other hand, COX-2, which was not expressed in U251MG cells, was not affected by any treatments (Figure 9D).

### 2.8. NS398 Counteracted TMZ-Induced MGMT Overexpression in T98G Cells

**Figure 10:**
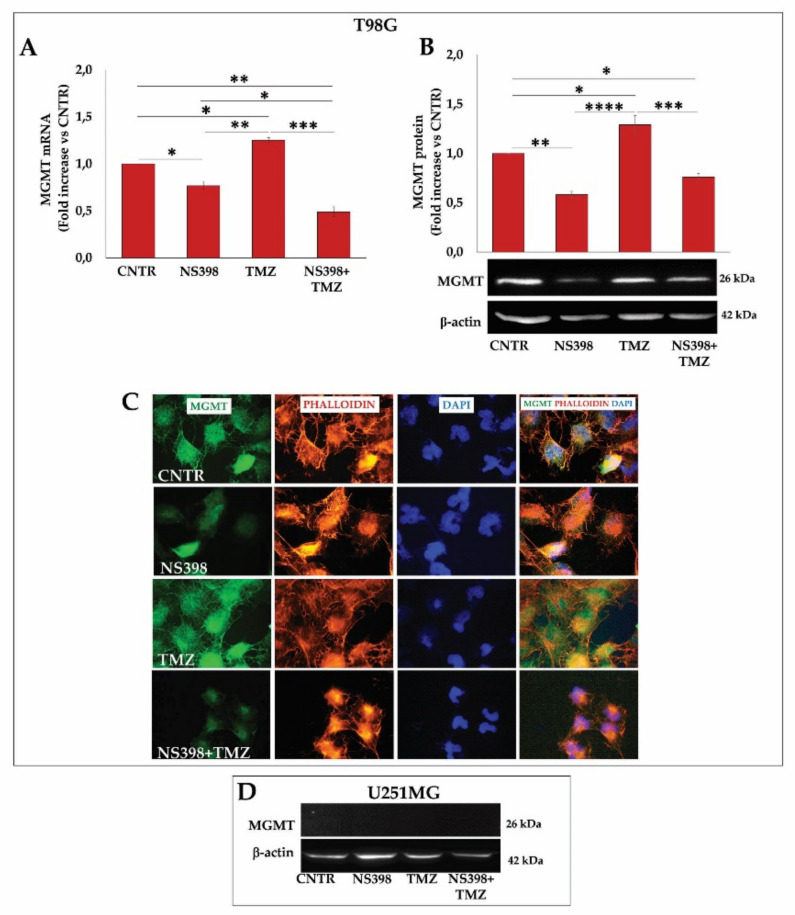
NS398 counteracted TMZ-induced MGMT overexpression in T98G cells. The cells were exposed to NS398 and TMZ, alone or in combination for 3 days, and MGMT levels were measured. (**A**) Relative mRNA expression levels of MGMT in TMZ-resistant T98G cell line are shown as fold increase vs. untreated cells (CNTR) (mean ± SEM; *n* = 2). (**B**,**D**) Immunoblotting assay for MGMT was performed on T98G and U251MG. Densitometric analysis is presented normalizing vs. β-actin. The data from three independent experiments are shown as the mean ± SEM and expressed as fold increase vs. CNTR. Images from one representative out of three independent experiments are shown. For comparative analysis of data, a one-way analysis of variance (ANOVA) with post hoc Tukey test was used (* *p* < 0.05; ** *p* < 0.01; *** *p* < 0.001; **** *p* < 0.0001). (**C**) Representative immunofluorescence images of T98G cells stained with anti-MGMT antibody (green) and with TRITC-phalloidin (red) to reveal F-actin from one out of three independent experiments are shown. Nuclei were counterstained with DAPI (blue). All images were acquired at 100× magnification.

To still investigate the ability of NS398 to counteract the TMZ resistance, the MGMT mRNA and protein levels were evaluated in the T98G cell line treated for 3 days with TMZ and NS398, alone or in combination. The COX-2 inhibitor significantly down-modulated, either at the transcriptional and post-transcriptional level, the basal and TMZ-induced MGMT expression (Figure 10A,B). These results were also confirmed by immunofluorescence analysis (Figure 10C). It is known that the U251MG are MGMT^-^ cells [42]; however, the effect of the different treatments on MGMT expression levels was evaluated. Both the single agents and the NS398+TMZ combination did not induce MGMT protein expression in these cells (Figure 10D).

### 2.9. NS398 Counteracted TMZ-Induced β-Atenin and SOX-2 Overexpression in T98G Cells

To further elucidate the mechanism underlying the NS398 ability in regulating the TMZ-resistance, we investigated the β-catenin expression involved in maintaining the stem-like state in GBM as well as in the progression and recurrence of the tumor [43]. The β-catenin protein expression in the T98G cell line exposed for 3 days to NS398 and TMZ, alone or in combination, was analyzed.

**Figure 11:**
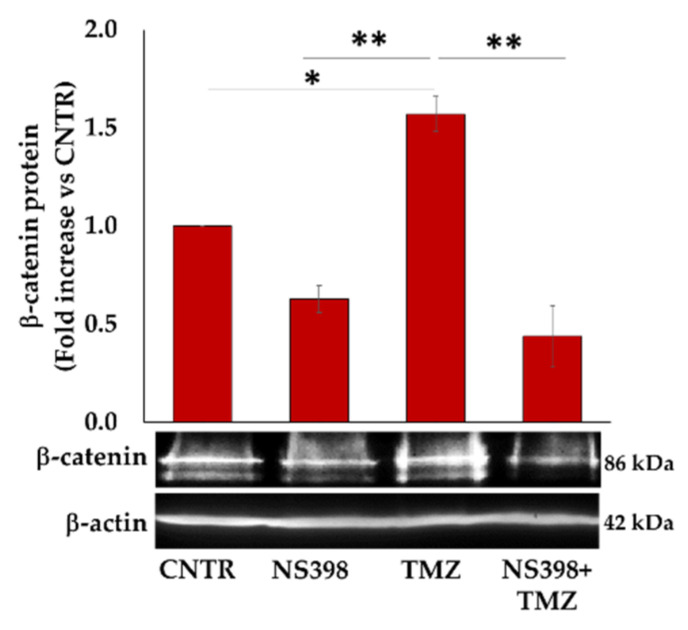
NS398 counteracted TMZ-induced β-catenin overexpression in T98G cells. The expression level of β-catenin was determined by WB in the T98G cells incubated for 3 days in the presence or absence (CNTR) of NS398 and TMZ, alone or in combination. The intensity of bands was quantified and normalized to β-actin, used as a loading control. Data shown are means ± SEM of three independent experiments and are expressed as fold increase vs. CNTR. Images from one representative out of three independent experiments are shown. For comparative analysis of data, a one-way analysis of variance (ANOVA) with Tukey post hoc test was used (* *p* < 0.05; ** *p* < 0.01).

Our results show that COX-2 inhibitor, NS398, down-regulated, even though not significantly, the β-catenin expression compared with CNTR, while TMZ alone significantly up-regulated its expression (Figure 11). Of interest, the treatment with NS398+TMZ counteracted the β-catenin expression induced by TMZ, considerably reducing it even below the baseline level.

**Figure 12:**
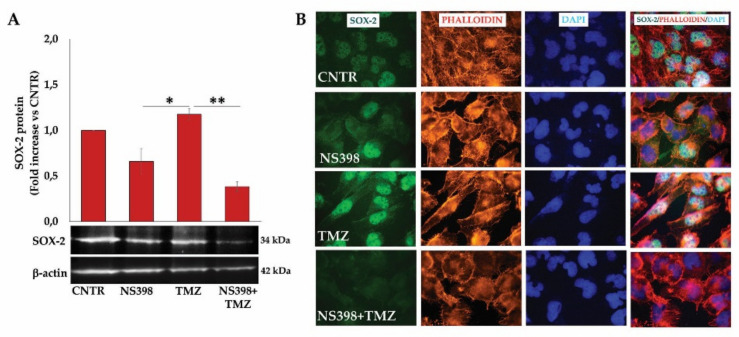
NS398 counteracted TMZ-induced SOX-2 overexpression in T98G cells. (**A**) Immunoblotting assay for SOX-2 was performed on T98G cells incubated for 3 days with NS398 and TMZ, alone or in combination. Following densitometric analysis, obtained values were normalized vs. β-actin. Data are from three independent experiments, and values (mean ± SEM) are expressed as fold increase vs. CNTR. Images from one representative out of three independent experiments are shown. For comparative analysis of data, a one-way ANOVA with post hoc Tukey test was used (* *p* < 0.05; ** *p* < 0.01). (**B**) Representative immunofluorescence images of T98G cells treated as above described and stained with anti-SOX-2 antibody (green) and with TRITC-phalloidin (red) to reveal F-actin, from one out of three independent experiments are shown. Nuclei were counterstained with DAPI (blue). All images were acquired at 100× magnification.

Finally, with the Wnt/β-catenin pathway being involved in GBM stem-like maintenance and considering the close link between GBM resistance and cancer stem cell presence, we wanted to analyze the effect of the COX-2 inhibitor alone or in combination with TMZ on the SOX-2 expression in T98G cells. As our group previously reported [44], we confirmed that T98G cells expressed high basal levels of SOX-2 stemness marker. The exposure to COX-2 inhibitor for 3 days slightly influenced the SOX-2 basal expression, although not significantly (Figure 12A). A huge increase in SOX-2 expression of T98G after TMZ treatment was detected, thus confirming previous data [45]. Notably, NS398 significantly counteracted the TMZ-induced SOX-2 expression (~3-fold decrease vs. TMZ alone) (Figure 12A). Representative images of immunofluorescence staining of T98G cells, showing the SOX-2 protein, localized mainly in nuclei, supported the above results (Figure 12B).

## 3. Discussion

Despite solid literature findings supporting the implication of COX-2 in TMZ resistance of GBM [21,24], as far as we know, there is no evidence that the drug of choice for the most common primary malignant brain tumor in adults can influence COX-2 expression and/or activity in GBM cells. Thus, the main objective of the present study was to evaluate the ability of the alkylating agent TMZ to influence COX-2 expression and activity in two GBM cell lines, i.e., T98G and U251MG, showing different sensitivity to TMZ, COX-2 expression level, and MGMT status.

Fully sharing what Herberer et al. have recently stated and proposed on the experimental use of TMZ in GBM research [46], with the aim of improving the transferability of our results to in vivo studies, we sought to take into account the available data on the pharmacokinetics of TMZ [47,48,49]. Thus, the TMZ concentration used for sensitive U251MG cells, as a single treatment, falls in the clinically relevant TMZ dose range (<35 µM). On the other hand, the proliferation rate and cell viability of TMZ-resistant T98G cells were not significantly affected at those concentrations. Thus, the chosen TMZ concentrations, able to keep the proliferation rate above 50%, were 200 µM for T98G cells and 10 µM for U251MG cells.

Our findings indicate that TMZ exposure of resistant T98G, but not of sensitive and COX-2 null U251MG cells, led to a relevant and dose-dependent up-regulation of COX-2, followed by a parallel increase in PGE2 generation. COX-2 expression is regulated by growth factors, proinflammatory cytokines including TNFα, IL-1β, IL-6, and Toll like receptors (TLRs). Accordingly, COX-2 promoter possesses a NF-κB (nuclear factor kappa-light-chain-enhancer of activated B cells) response element as well as other cytokines and growth factor response elements [50,51]. NF-κB signaling plays a crucial role in GBM, whose activation is, in turn, an important driver of the malignant phenotype, thus conferring a negative prognosis in GBM patients [52]. Receptor tyrosine kinases, mainly epidermal growth factor receptor (EGFR) and platelet derived growth factor receptor (PDGFR), which are often aberrantly activated in GBM, have been linked to NF-κB activation. Of note, TMZ exposure of T98G cells has been reported to increase the release of EGF and its receptor that, in turn, led to the expression of multidrug resistance gene (MDR1) [53]. Based on these data in the literature, our results showing the ability of TMZ to induce an up-regulation of COX-2 expression could be ascribed to the action exerted by the drug at the EGF/EGFR pair level leading to the NF-κB up-modulation and, hence, to an over-expression of COX2.

According to previous reports, TMZ is able to up-regulate Wnt/β-catenin signaling, MGMT expression, and stemness potential, all implicated in the GBM-chemoresistance [17,27,31]. We confirmed that in T98G cells, TMZ was able to increase β-catenin, MGMT, and SOX-2 expression, while no influence was recorded in U251MG. These results suggest that the effects on T98G cells are owing to TMZ-induced COX-2 up-regulation, which, in turn, is responsible for the over-activation of the Wnt/β-catenin pathway. MGMT and SOX-2 up-modulation appear both under the positive control of Wnt/β-catenin.

The specific COX-2 inhibitor, NS398, was used in our models to further investigate the role of the COX-2/PGE2 system in the TMZ resistance mechanisms of T98G cells.

First of all, the data obtained show that NS398, alone or combined with TMZ, influenced, in a more or less significant way, just COX-2^+^ T98G cells, while no effect was detected in U251MG, confirming that the NS398 activity was closely associated with COX-2 inhibition.

Surprisingly, the NS398, as expected, in addition to being effective in inhibiting the enzymatic activity of COX-2, was efficient in significantly reducing its gene and protein expression in T98G. This ability appears to be of particular interest when the inhibitor was combined with TMZ, being associated with the reduction in chemoresistance in these cells. According to our knowledge, this is the first evidence that the treatment with a COX-2 inhibitor leads to increased chemosensitivity to TMZ in GBM cells through a down-regulation of the overexpression of COX-2 levels, as well as through activity induced by the drug. A possible indirect mechanism could explain the observed effects, i.e., PGE2 could exert positive feedback on the COX-2 expression, and the inhibition of its synthesis by NS398 could weaken this mechanism.

Our findings also show that NS398 was able to overcome TMZ-induced overexpression of β-catenin, MGMT, and SOX-2 in T98G, thus confirming the key and the hierarchically superior role played by the COX-2/PGE2 system in the cascade of pathways activated by TMZ and implicated in chemoresistance. NS398 has been one of the earliest COX-2-selective inhibitors discovered [54] and, even if not yet approved by the FDA for clinical use, it continues to be largely used, also recently, by a plethora of researchers, as a prototype COX-2 inhibitor for in vitro and in vivo studies on several types of cancers, including GBM [20,36,55,56]. For our group, in particular, the present work represents a continuation of previous studies showing the ability of NS398 to influence the biology of GBM cell lines as well as the relative derived-neurospheres [13]. At the same time, pending pharmacokinetic studies and clinical trials with NS398, research activity is in progress to investigate the effect of other COXIBs, such as celecoxib, to verify the reproducibility of the effects obtained with NS398 on chemoresistance and to better elucidate the mechanisms involved.

Further studies are needed for a more complete analysis of the mechanisms underlying the interactions between the COX-2/PGE2/EP4 system and the Wnt/β-catenin pathway most involved in TMZ resistance. Another interesting aspect to study will be the expression of the target genes of Wnt signaling involved in the phenomena of chemoresistance of GBM.

One limitation of our study was the use of too short culture times that did not allow an evaluation of the long-term effects. More extended treatments could help to understand whether the restoration of chemosensitivity represents a long-lasting mechanism or, conversely, the possibility that the cell can activate additional mechanisms of resistance. Studies are in progress to address these aspects as well as to more directly characterize the players involved in the complex and intricate mechanism of TMZ resistance. To this aim, more GBM cell lines, as well as new approaches, are being evaluated, including COX-2-gene silencing in TMZ-resistant or -sensitive GBM cell lines, COX-2 transfection in COX-2 null GBM cells, and induction of TMZ-resistance in selected GBM cell lines. Moreover, considering the key role played by the COX-2/PGE2/EP signal pathway not only in GBM-chemoresistance but also in immunotherapy-resistance, the study of inhibition of this pathway in preclinical models could be of particular interest [57,58]. An innovative preclinical model particularly appealing in this context could be represented by GBM patient-derived xenografts (PDX) implanted in a humanized mouse model, immunodeficient mice with peripheral blood mononuclear cells derived from the same patient, recapitulating the patient’s immune-GBM interface [59,60].

## 4. Materials and Methods

### 4.1. Cell Lines and Culture Conditions

Human GBM grade IV cell lines, T98G and U251MG, were purchased from the European Collection of Authenticated Cell Cultures (ECACC) and Cell Lines Service (CLS), respectively, and manipulated according to the instructions given in the product sheet to maintain a stable genetic profile. The main characteristics of GBM cell lines are as follows: T98G cells express high levels of MGMT, p53 and PTEN are mutated, and p14ARF/p16 deleted; U251MG cells are p53 wild type, PTEN null, p14ARF/p16 deleted and MGMT null [38,61]. The basal expression of both MGMT and SOX-2 was also evaluated by Western blotting in T98G and U251MG (Supplementary Figure S1). The cell lines were grown as adherent cells in Dulbecco’s Modified Eagle’s Medium (DMEM) supplemented with 10% (*v*/*v*) of fetal calf serum (FCS), 2 mM L-glutamine, 100 U/mL penicillin, and 100 mg/mL streptomycin (complete medium) (EuroClone, West York, UK). As previously reported in Lee SY, the T98G and U251MG show a different sensitivity to TMZ: T98G cells are defined “TMZ-resistant”, showing a LC50 ranged from >250 mM to 1585 mM, and U251MG cells are considered “TMZ-sensitive”, showing a LC50 around 50 μM [38]. Cells were maintained at 37 °C in a humidified atmosphere of 95% air and 5% CO_2_, and medium was totally replaced every three days.

### 4.2. TMZ, and NS398 Preparation and Analysis of Cell Viability and Growth

The clinically used TMZ chemotherapy was obtained by Sigma-Aldrich (Saint Louis, MO, USA) prepared as stock solutions in sterile 100% dimethyl sulfoxide (DMSO), whose final concentration in the culture medium was 51.5 mM. This concentration did not influence cell viability and the expression of the studied proteins. Based on previous reports [62,63,64], TMZ at different final concentrations (10, 100, 200, and 400 µM) was tested by dose–response curve for 24, 48, and 72 h to select the drug concentration for the subsequent experiments. More precisely, the specific concentration of chemotherapeutic agent able to maintain the proliferation at 72 h of each cell line above 50% was chosen for the experiments designed to analyze the effects of combined treatment NS398+TMZ. The selective COX-2 inhibitor, NS398 (N-[2-(Cyclohexyloxy)-4-nitrophenyl]methanesulfonamide), was acquired from Sigma-Aldrich, and according to manufacturer instruction, the COX-2 inhibitor was stored as stock solutions in DMSO at −20 °C and diluted in cell culture medium just before use. The choice of NS398 concentration range (50–400 µM) and treatment times (up to 72 h) was first based on previous reports and in vitro studies on GBM cell lines [12,20]. Moreover, the NS398 concentration able to effectively inhibit COX-2 activity as well as proliferation rate of T98G cells was chosen for the subsequent experiments designed to analyze the effects of combined treatment NS398+TMZ. Analysis of viable and healthy cells treated with TMZ or NS398 was performed utilizing the IncuCyte^®^ Live Cell Imager system (Essen BioSciences, Inc., Ann Arbor, MI, USA). Briefly, cells were plated in a 96-multiwell culture plate at 1000 cells/cm^2^ and allowed to attach overnight. Once attached, media were replaced with media containing TMZ (10, 100, 200, and 400 µM) or NS398 (50, 100, 200, and 400 µM), and NucLight Rapid Red live-cell nuclear dye (1:4000 dilution), a lentivirus-based reagent that stably integrates into the host cell line without affecting cell growth or morphology, was added. Culture plates were sited into the IncuCyte^®^ Live Cell imager, and images were captured using phase contrast and red (400 msec exposure) channels and were taken every 4 h in the IncuCyte ZOOM™ platform (Essen BioSciences, Inc.). Four image sets were acquired from several points of the well, using a 10× objective lens, and all the treatment conditions were run in triplicates. At the end of the experiment, the proliferation rate, based on NucLight dye internalization, was analyzed by IncuCyte^®^ S3 2018C software (Essen BioSciences, Inc.). The treatment with the compound vehicle alone, DMSO, was referred to as “control” (CNTR).

To evaluate the effect of NS398, TMZ, and their combination on cell viability, GBM cell lines were plated for all experiments in 25 cm^2^ plates at 5000 cells/cm^2^, left to adhere overnight, and then treated with the COX-2 inhibitor, NS398, TMZ, and compounds combined at selected concentrations. The time of exposure to treatments was set for 3 days (72 h). Following treatment, the cells were harvested and centrifuged for 10 min at 400× *g*. Cell count was assessed in a Bürker chamber by optical microscopy (Eclipse 50i, Nikon, Tokyo, Japan) using Trypan blue solution (0.04%, final concentration, EuroClone, West York, UK). Not-treated cells, referred to as CNTR, were also analyzed. Cell morphology was evaluated by an optical microscope (Eclipse 50i, Nikon), and images were acquired at 10× and 20× magnification. The cells treated with different concentrations of TMZ were also used for COX-2 mRNA and protein quantification. All reagents for cell biology and consumables were purchased from EuroClone where not otherwise specified.

### 4.3. Total RNA Extraction and Gene Expression

To examine the basal expression of COX-2 in GBM cell lines, total RNA was isolated according to the RNeasy Mini Kit (QIAGEN, Hilden, Germany). RNA was spectrophotometrically quantified, and its quality was assessed by 1% agarose/Tris–Acetate–EDTA (TAE) gel electrophoresis. First-strand cDNA was synthesized using total RNA and the M-MLV cDNA Synthesis kit (Invitrogen, Waltham, MA, USA). Specific primers for COX-2 (forward 5′-ATTGTACCCGGACAGGATTCTATG-3′ and reverse 5′-TTTGGAGTGGGTTTCAGAAATAATT-3′) and β-actin (forward 5′-CACCATTGGCAATGAGCGGTTC-3′ and reverse 5′-AGGTCTTTGCGGATGTCCACGT-3′) were used to amplify gene fragments with the annealing temperature set at 60 °C. The PCR products were analyzed on 1.2% agarose gel and visualized by EuroSafe Nucleic Acid Staining (EuroClone, West York, UK). Densitometric analysis was performed using ImageJ software to quantify the band intensities. Data represent average values from three independent experiments.

Gene expression analysis of COX-2 and MGMT was performed in untreated (CNTR), treated NS398-, TMZ- and NS398+TMZ-T98G cells via real-time RT-PCR using a ViiA7 sequence detection system (Applied Biosystems, Foster City, CA, USA). Total RNA was extracted from cells using the RNeasy Mini Kit and quantified by spectrophotometry. An amount of 1 µg of total RNA was reverse transcribed in a final volume of 20 μL using a mixture of random primers below reported. An amount of 1 µg of each cDNA was used to perform the real-time PCR. Real-time quantitative RT-PCR analysis was carried out by SYBR Green dye detection (Thermo Fisher Scientific Waltham, MA, USA) according to the manufacturer’s instructions. Reverse and forward primers, purchased from IDT (acquired from Integrated DNA Technologies, IDT, Coralville, IA, USA), were used at a concentration of 1 µM (COX-2, MGMT, GAPDH), and their sequences were as follows: MGMT forward: 5′-GTCGTTCACCAGACAGGTGTTA-3′ and reverse 5′-ACAGGATTGCCTCTCATTGCTC-3′; COX-2 forward 5′-ATTGTACCCGGACAGGATTCTATG-3′ and reverse 5′-TTTGGAGTGGGTTTCAGAAATAATT-3′. In the T98G cells, as internal control for gene expression normalization, the mRNA level of GAPDH was measured by following primers: GAPDH forward 5′-TTGCCCTCAACGACCACTTT-3′ and reverse 5′-TGGTCCAGGGGTCTTACTCC-3′. The fold-change quantification of target genes was calculated with the 2^-∆∆Ct^ method [65]. The samples were run in triplicate, and the experiment was repeated twice.

### 4.4. Western Blot (WB) Analysis

Control and treated cells were harvested, washed in phosphate-buffered saline (PBS), and homogenized and lysed in ice-cold RIPA buffer (Merck KGaA, Darmstadt, Germany) containing 100 mM protease inhibitor cocktail (Sigma-Aldrich, Saint Louis, MO, USA). The protein amount was evaluated with DC Protein Assay (Bio-Rad, Hercules, CA, USA) using BSA as standard. An amount of 25 μg of protein lysates was mixed with sample buffer; samples were denatured (at 100 °C for 5 min) and run on 10% SDS polyacrylamide gel. Proteins were transferred onto 0.45 μm nitrocellulose membrane sheets (Bio-Rad) for 1 h at 4 °C at 70 V using a Mini Trans-Blot Cell apparatus (Bio-Rad). Non-specific binding sites were blocked with 5% non-fat dry milk for 1 h at room temperature and then incubated overnight at 4 °C with primary antibodies: rabbit monoclonal anti-COX-2 (Cell Signaling Technology, Danvers, MA, USA; dilution 1:1000), mouse monoclonal antibody anti-MGMT (BD Biosciences, San José, CA, USA; dilution 1:500), mouse monoclonal antibody anti-SOX2 (Origene, 9620 Medical Center Drive Suite 200 Rockville, MD, USA; dilution 1:1000), rabbit polyclonal antibody anti-β-catenin (Cell Signaling Technology, Danvers, MA, USA; dilution 1:1000), and mouse monoclonal antibody for anti-β-actin antibody (Bio-Rad, Hercules, CA, USA; dilution 1:1000). The horseradish peroxidase (HRP)-conjugated anti-mouse and anti-rabbit secondary antibodies were purchased from Sigma-Aldrich (Saint Louis, MO, USA). Immunoreactive bands were visualized by enhanced chemiluminescence (ECL, Amersham Pharmacia Biotech), according to the manufacturer’s instructions. Band relative densities were determined using a chemiluminescence documentation system ALLIANCE (UVITEC, Cambridge, UK), and values were given as relative units. All data were normalized to β-actin protein levels, used as internal control, and results are expressed as arbitrary units.

### 4.5. Prostaglandin 2 (PGE2) Level Assay

The supernatants of T98G cells were collected after treatment for 72 h with NS398, TMZ, and combination at the concentrations above indicated and then assayed for prostaglandin E2 (PGE2) levels by an enzyme-linked immunosorbent assay (ELISA) kit (Cayman Chemical Company, Ann Arbor, MI, USA) as described in the manufacturer’s instructions. The least detectable PGE2 concentration was 7.8 pg/mL. Results are presented as pg/mL of PGE2.

### 4.6. Cell Cycle Distribution and Apoptotic Cells Detection

The effects of COX-2 inhibitor and of TMZ on the cell cycle and apoptosis were examined in T98G and U251MG cell lines exposed to agents alone and their mixture for 3 days. At the end of incubation times, adherent cells were detached using trypsin–EDTA solution (EuroClone, West York, UK), counted, and fixed by using a cooled 70% ethanol solution (Sigma-Aldrich, Saint Louis, MO, USA), in PBS, with gentle mixing at 4 °C for 30 min. Fixed cells (10^5^ cells/mL) were transferred to plastic BD tubes (BD Biosciences, San José, CA, USA), washed twice with ice-cold PBS, and resuspended with a solution containing 50 μg/mL PI, 0.1% Nonidet-P40, and RNase A (6 μg/10^6^ cell) (all reagents acquired from Sigma-Aldrich, Saint Louis, MO, USA) for 30 min in the dark at 4 °C. The percentages of cells in the G1, G2/M, and S phases (data from 10,000 events per sample) were calculated by software Modfit LT for Mac V3.0 using a FACS Calibur instrument (BD Biosciences). The assay was carried out in duplicate. Apoptotic cells were determined by their hypochromic subdiploid nuclei staining. Data from 10,000 events per sample were collected and analyzed using a FACS Calibur instrument (BD Biosciences) equipped with cell cycle analysis software (Modfit LT for Mac V3.0).

### 4.7. Colony Formation Assay

The colony formation assay, or clonogenic assay, is an in vitro cell survival assay, a gold standard technique used for the study of chemical or anti-cancer agents, evaluating the cells’ ability to generate clones following treatment and allowing the evaluation of cytotoxic and/or antiproliferative effects [66]. Briefly, T98G and U251MG were seeded in 6-well plates at a concentration of 1000 cells/well and were incubated at 37 °C overnight for attachment. The next day, cell medium was changed, cells were treated with a single dose of NS398 (200 µM), TMZ (200 µM for T98G and 10 µM for U251MG), and their combination and placed in the CO_2_ incubator for 3 days. After this treatment, the cells were maintained in a fresh medium until colony formation attested on 20 and 18 days for T98G and U251MG cultures, respectively. Colonies were gently washed, fixed with cold methanol for 20 min, and stained with crystal violet 0.1% in PBS at room temperature for 10 min and air-dried. Colonies were visualized, and images were captured by optical microscopy (Eclipse 50i, Nikon, Tokyo, Japan). Colonies formed were counted using the ColonyCountJ, an add-on program, a semi-automated colony counting program to ImageJ, which provides the number of colonies [67]. The experiment set was carried out twice in duplicate, and histogram plots are the average number from three analyses.

### 4.8. Immunofluorescence Staining

The T98G cells were grown on coverslips in a 12-well plate (seeded at 3000 cells/cm^2^) and let to adhere overnight, then were treated with NS398, TMZ, and the combination for 3 days as previously reported. At the end of treatment, the coverslips were washed with PBS, fixed with 4% formaldehyde for 20 min, permeabilized with 0.1% Triton X-100 (Sigma-Aldrich, Saint Louis, MO, USA) for 5 min, and blocked with 3% BSA (Sigma-Aldrich) for 20 min at room temperature. Cells were incubated overnight at 4 °C with mouse monoclonal antibody anti-human MGMT (BD Biosciences, San José, CA, USA; dilution 1:500). Next, the coverslips were stained using a FITC conjugated goat anti-mouse polyclonal IgG secondary antibody (Bethyl Laboratories, Inc, Montgomery, TX, USA) for 1 h at room temperature and washed. For SOX-2 immunostaining, cells were incubated overnight at 4 °C with FITC-conjugated mouse monoclonal antibody anti-SOX2 (Origene, 9620 Medical Center Drive Suite 200 Rockville, MD, USA; dilution 1:100). All samples were then incubated with TRITC labeled phalloidin (Sigma-Aldrich) for 45 min at room temperature. The coverslips were mounted with VECTASHIELD^®^ Antifade Mounting Medium with DAPI (Vector Laboratories, Inc., Burlingame, CA, USA) and then examined at 100× magnifications with the fluorescent microscope (Eclipse 50i, Nikon, Tokyo, Japan).

### 4.9. Statistical Analysis

The GraphPad Prism 6.0 (GraphPad Software, San Diego, CA, USA) software was used for statistical analysis. Data were assessed for normality using the Shapiro–Wilk test and found to be normally distributed (*p* > 0.05). Comparisons between groups were performed using one-way or two-way ANOVA followed by Tukey or Dunnett’s post hoc test, where specified. In particular, the two-way for repeated measures ANOVA was used to evaluate the effect of two within-subject factors on a continuous outcome variable simultaneously. Data are expressed as the mean ± SEM (standard error mean) of independent experiments performed in duplicate or triplicate. Results were considered significant if *p* < 0.05.

## 5. Conclusions

Altogether, our results strongly support the role of the COX-2/PGE2 system in GBM TMZ-resistance. Moreover, as far as we know, this is the first evidence that TMZ induces COX-2 up-modulation in TMZ-resistant GBM cells. This effect was associated with the up-modulation of β-catenin, MGMT, and SOX-2 expression and well correlated with TMZ-resistance. COX-2 inhibition abrogated TMZ-induced COX-2 up-regulation and all COX-2-dependent pathways involved in TMZ-resistance. Although further studies are needed to gain a complete picture of the actors involved in the observed effects, overall, our data help to broaden the complex interplay of TMZ-resistance.

## Data Availability

The datasets generated and analyzed during the current study are available from the corresponding authors upon reasonable request.

## Supplementary Materials

The following supporting information can be downloaded at: https://www.mdpi.com/article/10.3390/ijms23031545/s1.
