# Supplementary material for: Up-Regulation of Cyclooxygenase-2 (COX-2) Expression by Temozolomide (TMZ) in Human Glioblastoma (GBM) Cell Lines
Source: Int J Mol Sci. 2022 Jan 28;23(3):1545. doi: 10.3390/ijms23031545 (PMC8835858; doi:10.3390/ijms23031545)
Supplement: Supplementary file 1 [file ijms-23-01545-s001.zip › ijms-1521988-supplementary.pdf]

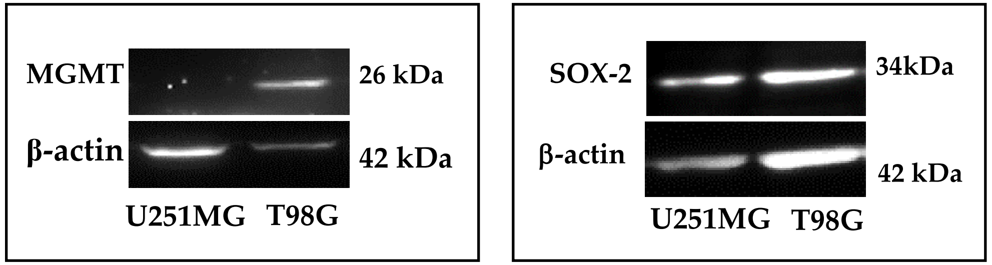

Figure S1. The O6-methylguanine DNA methyltransferase (MGMT) and SOX-2 basal expression in GBM cells. MGMT and SOX-2 levels were assayed in U251MG and T98G cell lines by Western blot. β-actin was used as the internal control. Images from one representative out of three independent experiments are presented.
